# Comparison of Oxidative Stress Markers with Clinical Data in Patients Requiring Anesthesia in an Intensive Care Unit

**DOI:** 10.3390/jcm13226979

**Published:** 2024-11-20

**Authors:** Fatih Segmen, Semih Aydemir, Onur Küçük, Cihangir Doğu, Recep Dokuyucu

**Affiliations:** 1 Department of Intensive Care Unit, Ankara City Hospital, Ankara 06800, Türkiye; drsegmen@gmail.com; 2 Department of Anesthesiology and Reanimation, Yenimahalle Training and Research Hospital, University of Yıldırım Beyazit, Ankara 06800, Türkiye; drsemihaydemir@gmail.com; 3 Department of Anesthesiology and Reanimation, Ankara Atatürk Sanatorium Training and Research Hospital, University of Health Sciences, Ankara 06290, Türkiye; dr.okucuk@gmail.com; 4 Department of Anesthesiology and Reanimation, Ankara Bilkent City Hospital Department of Intensive Carei, Ankara 06800, Türkiye; cihangirdogu@gmail.com; 5 Department of Physiology, Medical Specialization Training Center (TUSMER), Ankara 06800, Türkiye

**Keywords:** intensive care unit, oxidative stress index, ferritin, C-reactive protein, procalcitonin, immature granulocyte, zinc, copper

## Abstract

**Objectives:** The aim of this study is to assess the oxidative stress status in patients requiring intensive care unit (ICU) admission before initiating ICU treatment, by measuring the total oxidant level (TOS) and total antioxidant level (TAS) and oxidative stress index (OSI) levels. Additionally, we aim to explore the correlation between these oxidative stress markers and biochemical and hematological parameters. **Materials and Methods:** A total of 153 patients treated in intensive care units were included in the study. Patients who met the patient admission criteria of the ethics committee of the intensive care medicine association were included in the study. Blood samples were taken at the first moment the patients were admitted to the intensive care unit (before starting treatment). In total, 60 healthy volunteers who were compatible with the patient group in terms of age and gender were included in the study as a control group. Patients who had previously received antioxidant treatment and cancer patients were excluded from the study. **Results:** The TOS was significantly higher in the patient group (13.4 ± 7.5) compared to controls (1.8 ± 4.4) (*p* = 0.021). TOS > 12.00 means a “very high oxidant level”. OSI was significantly higher in the patient group (689.8 ± 693.9) compared to the control group (521.7 ± 546.6) (*p* = 0.035). Ferritin levels were significantly higher in the patient group (546.5 ± 440.8 ng/mL) compared to controls (45.5 ± 46.5 ng/mL) (*p* < 0.001). Patients had significantly higher levels of C-reactive protein (CRP), procalcitonin (PCT), white blood cells (WBCs), immature granulocytes (IGs), zinc, and copper compared to the control group, indicating elevated inflammation and oxidative stress. CRP levels were 76.6 ± 85.9 mg/L in patients versus 5.6 ± 15.1 mg/L in controls (*p* < 0.001). PCT levels were 15.8 ± 8.6 ng/L in patients versus 2.3 ± 7.2 ng/L in controls (*p* = 0.012). Zinc and copper were also significantly elevated (*p* = 0.012 and *p* = 0.002, respectively). **Conclusions:** Our study provides valuable insights into the relationship between oxidative stress, inflammation, and trace elements, contributing to the growing understanding of oxidative stress as a prognostic tool in critical care. This could help to tailor therapeutic strategies aimed at reducing oxidative damage in ICU patients, enhancing patient outcomes.

## 1. Introduction

Oxidative stress, characterized by an imbalance between the production of reactive oxygen species (ROS) and the body’s antioxidant defenses, plays a crucial role in the pathophysiology of critical illnesses [1,2,3]. In the intensive care unit (ICU), patients often exhibit elevated levels of oxidative stress due to various underlying conditions such as sepsis, trauma, and acute respiratory distress syndrome. This oxidative imbalance can lead to cellular damage and organ dysfunction, contributing to the severity of the patient’s condition [4,5].

The total antioxidant status (TAS) and total oxidative status (TOS) are valuable markers that provide insight into the overall oxidative environment within the body. TAS reflects the cumulative action of all antioxidants present in plasma, whereas TOS measures the total amount of oxidants, including free radicals and peroxides [6]. The oxidative stress index (OSI), which is the ratio of TOS to TAS, is a widely used parameter to quantify the oxidative stress burden [7].

In patients requiring intensive care, various physio-pathological processes have been associated with the unregulated production and release of large amounts of reactive oxy-gen species (ROS) [8]. Oxidative stress may be a primary factor in disease. However, once damage has begun, antioxidant therapy often fails to prevent the progression of tissue damage because other factors become dominant in the pathology [9]. Therefore, determining the oxidative stress status is important for the effectiveness of treatment.

Oxidative stress plays an important role in the pathophysiology of critical illnesses. In conditions such as sepsis, acute respiratory distress syndrome (ARDS), and multiple organ failure, reactive oxygen species (ROS) are excessively produced and cause destructive effects on cellular structures. This situation triggers organ dysfunction by increasing the inflammatory response and negatively affects the prognosis of patients. It is known that oxidative stress accelerates mitochondrial dysfunction and suppresses the immune response, especially in patients with sepsis. In addition, high levels of oxidative stress markers (TOS, OSI) are closely associated with mortality and complications in patients hospitalized in the intensive care unit (ICU). Therefore, the early identification and management of oxidative stress has an important role in shaping treatment strategies in critically ill patients [1,3,5,9].

Oxidative stress markers are increasingly recognized as valuable tools in evaluating the physiological state of critically ill patients. These markers, when correlated with clinical parameters such as ferritin, C-reactive protein (CRP), procalcitonin (PCT), and components of the complete blood count (CBC), provide a comprehensive picture of the inflammatory and infectious processes at play. Ferritin, an acute-phase reactant, not only reflects iron storage but also serves as a marker of systemic inflammation, often elevated in critical conditions like sepsis and MODS. Similarly, CRP and PCT are well-established biomarkers for detecting and monitoring inflammation and bacterial infections, respectively. Elevated levels of CRP and PCT in critically ill patients have been associated with worse outcomes, highlighting their prognostic value. Additionally, CBC components such as the white blood cell (WBC) count and immature granulocyte (IG) levels offer insights into the immune response and bone marrow activity under stress [1,10,11,12].

The aim of this study is to assess the oxidative stress status in patients requiring ICU admission before initiating ICU treatment, by measuring TAS, TOS, and OSI levels. Additionally, we aim to explore the correlation between these oxidative stress markers and various clinical parameters such as ferritin, CRP, PCT, and WBC components, which are commonly used to assess inflammation and infection in critically ill patients.

## 2. Materials and Methods

### 2.1. Study Design and Study Population

Informed consent was obtained from all participants and the study was conducted between January 2023 and January 2024 at City Hospital. A total of 153 patients treated in intensive care units were included in the study. As inclusion criteria, patients were those aged 18 years and older, admitted to the ICU with conditions such as sepsis, acute respiratory distress syndrome (ARDS), or multiple organ dysfunction syndrome (MODS), and who provided informed consent. Patients who met the patient admission criteria of the ethics committee of the intensive care medicine association were included in the study [13]. Blood samples were taken at the first moment the patients were admitted to the intensive care unit (before starting treatment). In total, 60 healthy volunteers who were compatible with the patient group in terms of age and gender were included in the study as a control group. As exclusion criteria for both groups, excluded patients were those with a history of antioxidant supplementation within the past three months, known malignancies, chronic inflammatory diseases, immune deficiency, or those receiving chemotherapy or radiotherapy. Additionally, patients with incomplete medical records or who withdrew consent during the study were excluded.

Total oxidant level (TOS) and total antioxidant level (TAS) measurements were performed using the methods described in the literature by Erel et al. [14,15]. TOS was determined by measuring the oxidation of a ferrous ion to a ferric ion in the presence of various oxidants in the sample. The resulting ferric ion forms a colored complex with xylenol orange, and the intensity of the color is proportional to the total amount of oxidants present. TAS was measured by assessing the ability of antioxidants in the sample to reduce ABTS•+ (2,2′-azinobis [3-ethylbenzothiazoline-6-sulfonic acid] radical cation), a stable blue-green chromophore. The decolorization rate of ABTS•+ is directly proportional to the total antioxidant capacity of the sample. Both measurements were performed using a Roche Cobas 6000 biochemistry analyzer, which ensures high precision and reproducibility. The TOS and TAS results were expressed in micromoles of hydrogen peroxide equivalent per liter (μmol H_2_O_2_ Eq/L) and micromoles of Trolox equivalent per liter (μmol Trolox Eq/L), respectively. The oxidative stress index (OSI) was calculated as the ratio of TOS to TAS, providing a comprehensive assessment of oxidative balance in the body.

Copper (Cu) and Zinc (Zn) levels were automatically analyzed using serum obtained from blood samples using a Roche Cobas 6000 c biochemistry analyzer (Roche Diagnostics, Basel, Switzerland). This device is a fully automatic system capable of high accuracy and reliability and is widely used to determine the concentrations of copper and zinc elements in the body.

Other biochemical and hematological parameters such as procalcitonin (PCT), ferritin, C-reactive protein (CRP), white blood cells (WBCs), and immature granulocytes (IGs) were obtained from the hospital information management system during the clinical follow-up of the patients. These data were used to evaluate the systemic inflammatory responses and the presence of infection in the study. Procalcitonin is a biomarker used to evaluate the presence of infections, while CRP is a general indicator of inflammation. White blood cells (WBCs) and immature granulocytes (IGs) were examined as markers of the inflammatory response and immunological activity in the bone marrow.

### 2.2. Statistical Analysis

Statistical analyses were performed using SPSS v.27 (IBM, Addison, TX, USA). The conformity of the data to a normal distribution was assessed using the Kolmogorov–Smirnov test. A chi-square test and independent groups *t* test (unpaired *t* test) were used to compare the data. Correlation analysis was performed using Pearson correlation analysis. A *p* value of <0.05 was considered significant for statistical significance.

## 3. Results

**Figure 1:**
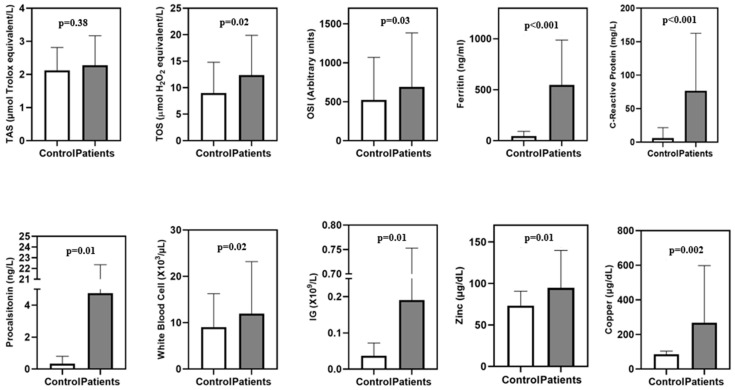
Comparison of biochemical and hematological parameters between the groups.

The TAS, TOS, OSI, and biochemistry values of the controls and patients are shown in Table 1. There was no statistically significant difference in age (*p* = 0.290) or gender distribution (*p* = 0.185) between the control and patient groups. In terms of diseases, there was no statistically significant difference between the groups (*p* > 0.05). No significant difference was observed in TAS between controls (2.13 ± 0.69) and patients (2.27 ± 0.89) (*p* = 0.380). The TOS was significantly higher in the patient group (13.4 ± 7.5) compared to controls (1.8 ± 4.4) (*p* = 0.021). TOS > 12.00 means a “very high oxidant level” (kit manufacturer information). OSI was significantly higher in the patient group (689.8 ± 693.9) compared to the control group (521.7 ± 546.6) (*p* = 0.035). Ferritin levels were significantly higher in the patient group (546.5 ± 440.8 ng/mL) compared to controls (45.5 ± 46.5 ng/mL) (*p* < 0.001). CRP levels were significantly higher in the patient group (76.6 ± 85.9 mg/L) compared to the control group (5.6 ± 15.1 mg/L) (*p* < 0.001). PCT levels were significantly higher in the patient group (15.8 ± 8.6 ng/L) compared to the control group (2.3 ± 7.2 ng/L) (*p* = 0.012). The WBC count was significantly higher in the patient group (18.0 ± 5.9 × 10^3^/μL) compared to the control group (7.9 ± 2.0 × 10^3^/μL) (*p* = 0.025). IG counts were also higher in the patient group (0.19 ± 0.56 × 10⁹/L) compared to the control group (0.04 ± 0.04 × 10⁹/L) (*p* = 0.013). Zinc levels were significantly higher in the patient group (86.1 ± 21.0 µg/dL) compared to the control group (66.5 ± 15.9 µg/dL) (*p* = 0.012). Copper levels were also significantly elevated in the patient group (243.7 ± 90.2 µg/dL) compared to the control group (77.2 ± 17.2 µg/dL) (*p* = 0.002) (Table 1, Figure 1).

Correlation analyses of patients’ TAS, TOS, OSI, and biochemical and hematological parameters are shown in Table 2. Statistical analysis revealed no significant correlation between the patient’s gender and oxidative stress markers (TOS, TAS, OSI) or clinical parameters such as ferritin, CRP, and PCT (*p* > 0.05). Ferritin showed a moderate negative correlation with TAS (r = −0.390, *p* = 0.001) and a strong positive correlation with both TOS (r = 0.670, *p* = 0.014) and OSI (r = 0.640, *p* = 0.011), indicating that increased ferritin levels are associated with reduced antioxidant capacity and elevated oxidative stress. CRP was moderately negatively correlated with TAS (r = −0.250, *p* = 0.034) and positively correlated with both TOS (r = 0.510, *p* = 0.001) and OSI (r = 0.680, *p* = 0.001), suggesting a link between systemic inflammation and increased oxidative stress. For PCT, a negative correlation was found with TAS (r = −0.320, *p* = 0.010), while positive correlations were seen with TOS (r = 0.485, *p* = 0.012) and OSI (r = 0.552, *p* = 0.013), indicating that elevated PCT is associated with higher oxidative stress. The WBC count demonstrated a weak negative correlation with TAS (r = −0.270, *p* = 0.025) and moderate positive correlations with TOS (r = 0.385, *p* = 0.016) and OSI (r = 0.412, *p* = 0.012), showing a relationship between increased WBC count and oxidative stress. IG counts showed a weak negative correlation with TAS (r = −0.202, *p* = 0.040) and moderate positive correlations with TOS (r = 0.268, *p* = 0.032) and OSI (r = 0.375, *p* = 0.027), reflecting a connection between elevated IG levels and oxidative stress. Trace elements, including Zn and Cu, showed significant correlations with oxidative stress markers. Zinc had a weak positive correlation with TAS (r = 0.290, *p* = 0.031) and moderate negative correlations with TOS (r = −0.437, *p* = 0.011) and OSI (r = −0.398, *p* = 0.002). Similarly, copper had weak positive correlations with TAS (r = 0.301, *p* = 0.034) and moderate negative correlations with TOS (r = −0.431, *p* = 0.020) and OSI (r = −0.407, *p* = 0.001), suggesting that both zinc and copper may contribute to reducing oxidative stress. In summary, ferritin, CRP, PCT, WBC, and IG all show positive correlations with oxidative stress markers (TOS and OSI), while being negatively correlated with TAS, indicating that higher levels of these parameters are associated with increased oxidative stress and a reduced antioxidant capacity. Zinc and copper, on the other hand, show an inverse relationship with oxidative stress markers (TOS and OSI), indicating their role in reducing oxidative stress and improving antioxidant capacity (TAS) (Table 2).

## 4. Discussion

The findings of this study demonstrate significant relationships between oxidative stress markers (TAS, TOS, OSI) and various biochemical and hematological parameters, including ferritin, CRP, PCT, WBC, and IG count, as well as trace elements like zinc and copper. These results align with and extend upon previous research in several important ways.

In this study, a negative correlation was observed between ferritin levels and TAS (r = −0.390, *p* = 0.001). In contrast, ferritin was positively correlated with TOS (r = 0.670, *p* = 0.014) and OSI (r = 0.640, *p* = 0.011). This indicates that higher ferritin levels, which are markers of inflammation and iron storage, are associated with increased oxidative stress. Similar results have been reported in studies of critically ill patients and those with chronic diseases, where elevated ferritin is commonly linked to oxidative stress and inflammation [16,17]. A study by Cakirca et al. found that elevated ferritin levels, a marker of inflammation, were associated with increased TOS and OSI, indicating higher oxidative stress in these patients. Conversely, ferritin showed a negative correlation with TAS, suggesting that as ferritin levels rise, the body’s antioxidant capacity decreases. This reflects the role of oxidative stress in the inflammatory response seen in COVID-19 [18]. In a study by Okuyan et al., a significant positive correlation was found between ferritin levels and oxidative stress markers, such as TOS and OSI, in chronic kidney disease patients. Additionally, ferritin was negatively correlated with TAS, indicating that higher ferritin levels are associated with increased oxidative stress and reduced antioxidant capacity in chronic kidney disease (CKD) patients. These findings suggest that ferritin may play a role in oxidative stress and inflammation in the progression of CKD [17]. These correlations suggest that ferritin could serve as a useful marker for assessing oxidative stress in ICU patients, complementing its established role in inflammation.

In our study, CRP exhibited a moderate inverse relationship with TAS (r = −0.250, *p* = 0.034) and a positive correlation with both (TOS (r = 0.510, *p* = 0.001) and OSI (r = 0.680, *p* = 0.001). CRP is a well-established marker of systemic inflammation, and several studies have demonstrated similar correlations, particularly in conditions such as sepsis and cardiovascular diseases, where oxidative stress plays a crucial role [10,19]. In a study by Akyuva et al., a relationship was reported between CRP and oxidative stress markers in patients with head and multiple organ trauma. It was found that CRP levels were positively correlated with liver enzymes and oxidative markers. The changes in CRP and oxidative stress markers suggest that CRP may serve as a useful indicator of oxidative stress and inflammation in trauma patients [20]. The strong positive relationship between CRP and OSI in particular highlights the close link between inflammation and oxidative imbalance in critically ill patients.

The correlations observed between PCT and oxidative stress markers further emphasize the role of oxidative stress in infection and systemic inflammation. In our study, PCT demonstrated a negative correlation with TAS (r = −0.320, *p* = 0.010), while showing a positive relationship with both TOS (r = 0.485, *p* = 0.012) and OSI (r = 0.552, *p* = 0.013). In a study by Cakir et al., a relationship was reported between PCT and oxidative stress markers in an experimental sepsis model. The results show that after treatment with fluoxetine, imipenem, and a combination of both, PCT levels, along with oxidative stress markers such as OSI and disulfide levels, were significantly decreased. They suggested that PCT may be associated with oxidative stress in the context of sepsis [18]. PCT is a recognized marker of bacterial infection, and elevated levels have been associated with increased oxidative stress in septic patients [21]. The current findings are consistent with studies that suggest PCT and oxidative stress markers like TOS and OSI can be used together to assess the severity of infections in ICU patients [11,22].

The results regarding WBC and IG counts also support the notion that oxidative stress is closely linked to the immune response [23]. Both WBC and IG showed positive correlations with TOS and OSI, and negative correlations with TAS. This is consistent with findings in other studies, where increased oxidative stress is observed alongside elevated immune cell activity, particularly in infections and inflammatory conditions [24,25,26]. In a study by Cetin et al., an association was found between WBC, IG, and oxidative stress markers in pediatric patients with CKD. It was found that higher levels of WBC and IG were significantly correlated with increased oxidative stress markers, including TOS and OSI, suggesting that IG levels may reflect systemic inflammation and oxidative stress in CKD patients [12]. The correlations observed in our study suggest that WBC and IG counts could serve as additional markers of oxidative stress in critically ill patients, particularly in the context of infection and systemic inflammation.

The role of trace elements such as zinc and copper in modulating oxidative stress was also evident in our study. Zinc and copper were both positively correlated with TAS and negatively correlated with TOS and OSI, suggesting their potential protective role against oxidative stress. This is consistent with the known antioxidant properties of these trace elements, particularly zinc, which has been shown to enhance antioxidant defense mechanisms by acting as a co-factor for antioxidant enzymes such as superoxide dismutase (SOD) [27]. Copper, on the other hand, plays a more complex role in oxidative stress, as it is both a co-factor for antioxidant enzymes and can promote oxidative damage in excess [28]. The negative correlations between copper and TOS/OSI in this study suggest that, within normal physiological levels, copper may help to reduce oxidative stress in critically ill patients. In a study of Pincemail et al., it was found that alterations in zinc and copper levels are closely associated with oxidative stress in patients hospitalized in the ICU for severe pneumonia. Specifically, copper is often linked to increased oxidative stress due to its role in the production of reactive oxygen species (ROS), while zinc tends to have an antioxidant effect, helping to reduce oxidative damage. These findings underscore the importance of maintaining balanced levels of these trace elements for oxidative stress regulation [29].

### Limitations of the Study

There were some limitations of the current study. One limitation of this study is that it did not account for long-term oxidative stress changes following ICU admission, as data were collected only at the point of admission. Additionally, the exclusion of patients with prior antioxidant therapy or cancer may limit the generalizability of the results to broader ICU populations. The strength of this study lies in its detailed analysis of the correlations between oxidative stress markers (TAS, TOS, OSI) and clinical parameters such as ferritin, procalcitonin, and immature granulocytes (IGs) in ICU patients, an area that has not been extensively explored in previous research. While oxidative stress has been widely studied in various diseases, its specific relationship with these clinical markers in critically ill patients provides novel insights into the role of oxidative stress in ICU settings.

## 5. Conclusions

In conclusion, the findings of this study underscore the close relationship between oxidative stress, inflammation, and trace elements in ICU patients. Ferritin, CRP, PCT, WBC, and IG all showed positive correlations with oxidative stress markers (TOS and OSI) and negative correlations with TAS, suggesting that these parameters may serve as valuable indicators of oxidative stress in critically ill patients. Importantly, the observed correlations highlight the potential of these markers, particularly TOS, OSI, and TAS, as prognostic tools for predicting clinical outcomes in the ICU setting. Zinc and copper, on the other hand, appear to play protective roles by enhancing the antioxidant capacity, potentially mitigating oxidative damage and improving patient outcomes. The ability to monitor oxidative stress and its associated biomarkers could aid in early risk stratification and guide targeted therapeutic interventions, such as antioxidant supplementation, to improve prognosis. The study’s findings contribute to the growing understanding of oxidative stress as a prognostic tool in critical care, emphasizing the need for further research to establish standardized thresholds for these markers and validate their predictive value in larger, multicenter cohorts. This could ultimately help to refine treatment strategies and improve patient outcomes in ICU settings.

## Figures and Tables

**Table 1:** TAS, TOS, OSI, and biochemistry values of controls and patients.

	Control (n = 60)	Patients (n = 93)	*p* Value
Age	58.3 ± 16.3	61.8 ± 21.6	0.290
Gender			0.185
Male	28	52	
Female	32	41	
Cardiovascular disease, n (%)	15 (25%)	24 (25.8%)	0.900
Respiratory system disease, n (%)	10 (16.6%)	18 (19.3%)	0.780
Diabetes, n (%)	8 (13.3%)	14 (15%)	0.750
Hypertension, n (%)	17 (28.3%)	30 (32.2%)	0.680
Dyslipidemia, n (%)	12 (20%)	22 (23.6%)	0.640
TAS (µmol Trolox equivalent/L)	2.13 ± 0.69	2.27 ± 0.89	0.380
TOS (μmol H_2_O_2_ equivalent/L)	1.8 ± 4.4	13.4 ± 7.5	0.021
OSI (Arbitrary units)	521.7 ± 546.6	689.8 ± 693.9	0.035
Ferritin (ng/mL)	45.5 ± 46.5	546.5 ± 440.8	<0.001
CRP (mg/L)	5.6 ± 15.1	76.6 ± 85.9	<0.001
PCT (ng/L)	2.3 ± 7.2	15.8 ± 8.6	0.012
WBC (×10^3^/µL)	7.9 ± 2.0	18.0 ± 5.9	0.025
IG (×10^9^/L)	0.04 ± 0.04	0.19 ± 0.56	0.013
Zn (μg/dL)	66.5 ± 15.9	86.1 ± 21.0	0.012
Cu (μg/dL)	77.2 ± 17.2	243.7 ± 90.2	0.002

TAS: Total antioxidant status, TOS: Total oxidant status, OSI: Oxidative stress index, CRP: C-reactive protein, PCT: Procalcitonin, WBC: White blood cell, IG: Immature granulocyte, Zn: Zinc, Cu: Copper.

**Table 2:** Correlation analysis of patients’ TAS, TOS, OSI, and biochemical and hematological parameters.

		TAS	TOS	OSI
Ferritin	r	−0.390	0.670	0.640
	*p*	0.001	0.014	0.011
C-reactive protein (CRP)	r	−0.250	0.510	0.680
	*p*	0.034	0.001	0.001
Procalcitonin (PCT)	r	−0.320	0.485	0.552
	*p*	0.010	0.012	0.013
White blood cell (WBC)	r	−0.270	0.385	0.412
	*p*	0.025	0.016	0.012
Immature granulocyte (IG)	r	−0.202	0.268	0.375
	*p*	0.040	0.032	0.027
Zinc (Zn)	r	0.290	−0.437	−0.398
	*p*	0.031	0.011	0.002
Copper (Cu)	r	0.301	−0.431	−0.407
	*p*	0.034	0.020	0.001

TAS: Total antioxidant status, TOS: Total oxidant status, OSI: Oxidative stress index. Statistically significant difference. r < 0.2: Indicates either no relationship or an extremely weak one. r = 0.2–0.4: Suggests a weak correlation between the variables. r = 0.4–0.6: Represents a moderate level of correlation. r = 0.6–0.8: Indicates a strong correlation.

## Data Availability

The original contributions presented in the study are included in the article, further inquiries can be directed to the corresponding author.
